# Early Vascular Ageing in adolescents with migraine with aura: a community-based study

**DOI:** 10.1186/s12872-023-03409-2

**Published:** 2023-08-01

**Authors:** Benoît Bernar, Nina Gande, Anna Katharina Stock, Anna Staudt, Raimund Pechlaner, Christoph Hochmayr, Katharina Kaltseis, Bernhard Winder, Sophia Zollner-Kiechl, Gregor Broessner, Ralf Geiger, Stefan Kiechl, Early Vascular Ageing (EVA) Tyrol Study Group, Benoît Bernar, Nina Gande, Anna Katharina Stock, Anna Staudt, Raimund Pechlaner, Christoph Hochmayr, Katharina Kaltseis, Bernhard Winder, Sophia Zollner-Kiechl, Gregor Broessner, Ralf Geiger, Stefan Kiechl, Markus Anliker, Mandy Asare, Manuela Bock-Bartl, Maximilian Bohl, Andrea Griesmacher, Julia Klingenschmid, Martina Kothmayer, Julia Marxer, Maximilian Pircher, Carmen Reiter, Christina Schreiner, Ursula Kiechl-Kohlendorfer, Michael Knoflach

**Affiliations:** 1 Department of Pediatrics, Pediatrics I, Medical University of Innsbruck, Innsbruck, Austria; 2 Department of Neurology, Medical University of Innsbruck, Anichstraße 35, Innsbruck, 6020 Austria; 3 Department of Pediatrics, Pediatrics II, Medical University of Innsbruck, Innsbruck, Austria; 4 Department of Pediatrics, Pediatrics III, Medical University of Innsbruck, Innsbruck, Austria; 5 Academic Teaching Hospital, Landeskrankenhaus Feldkirch, Feldkirch, Austria; 6 VASCage, Research Centre on Vascular Ageing and Stroke, Innsbruck, Austria; 7 Department of Neurology, Hochzirl-Natters Hospital, Zirl, Austria

**Keywords:** Headache, Migraine with aura, Early vascular ageing, Pulse-wave velocity, Carotid intima-media-thickness, Adolescents, EVA-Tyrol study

## Abstract

**Background:**

Migraine with aura is associated with an increased risk of cardiovascular disease, yet the pathophysiology is unknown. Suggested underlying mechanisms of aura formation point into the direction of an abnormal vasoreactivity that also extends to the extracranial vasculature.

**Methods:**

In the Early Vascular Ageing Tyrol study, a community-based non-randomized controlled trial conducted in 45 schools and companies in Tyrol (Austria) and South-Tyrol (Italy) between May 2015 and September 2018 aiming to increase cardiovascular health in adolescents, headache syndromes were classified according to the International Classification of Headache Disorders in a face-to-face interview. Carotid-femoral pulse-wave-velocity was measured by applanation tonometry and carotid intima-media-thickness by high-resolution ultrasound of the distal common carotid arteries. Differences in pulse-wave-velocity and carotid intima-media-thickness in youngsters with migraine with aura were compared respectively to those without headache and with other headaches by multivariable linear regression analysis.

**Results:**

Of the 2102 study participants 1589 were aged 14 to 19 (mean 16.8) years and had complete data. 43 (2.7%) reported migraine with aura and 737 (46.4%) other headaches. Mean pulse-wave-velocity was 6.17 m/s (± 0.85) for migraine with aura, 6.06 m/s (± 0.82) for all other headaches and 6.15 (0.95) m/s for participants without headaches. Carotid intima-media-thickness was 411.3 µm (± 43.5) for migraine with aura, 410.9 µm (± 46.0) for all other headaches and 421.6 µm (± 48.4) for participants without headaches. In multivariable linear regression analysis, we found no differences in carotid-femoral pulse-wave-velocity or carotid intima-media-thickness in young subjects with migraine with aura, all other headaches, or no headaches.

**Conclusions:**

In line with previous large-scale studies in adults, we could not demonstrate relevant associations of migraine with aura with markers of arterial stiffness or subclinical atherosclerosis making early vascular ageing an unlikely pathophysiological link between migraine with aura and cardiovascular diseases.

**Trial registration:**

First registered on ClinicalTrials.gov 29/04/2019 (NCT03929692).

**Supplementary Information:**

The online version contains supplementary material available at 10.1186/s12872-023-03409-2.

## Introduction

According to the World Health Organization (WHO) half of the adult population is affected by recurrent headaches [1] and the lifetime prevalence for headache is close to 100% [2]. The prevalence of migraine is up to 21.7% in female and in 11.3% in male students [3]. Migraine is accompanied by aura in a third to a fifth of the patients [4]. The burden of disease of headache disorders is high and migraine is one of the five leading causes of years lived with disability [5]. Besides the direct burden of disease, migraine with aura (MA) is related to an increased risk of cardiovascular diseases (CVD) [6] and a higher CVD mortality [7]. MA increases the risk of stroke or transient ischemic attack (TIA) by a factor of two in the general population [4, 6] and triples the risk among women younger than 45 years [8, 9]. Furthermore, an ischemic stroke as a consequence (or cause) of a migrainous attack with aura (migrainous infarction) accounts for about 13% of the first ischemic stroke of unusual etiology in young adults [10]. Even though the association between MA and CVD is firmly established on an epidemiological basis, the pathophysiological pathway is a matter of ongoing discussions. Suggested underlying mechanisms of aura formation like cortical spreading depression, vasodilation or trigeminal-dysfunction point in the direction of an abnormal vasoreactivity that also extends to the extracranial vasculature [11].

It is a well-established fact, that atherosclerosis, the predominant cause of CVD, starts early in life [12–14]. Early vessel wall changes can already be demonstrated in children and adolescents with chronic diseases [15–17]. Due to continuous vascular ageing and accelerated by cardiovascular risk factors, arterial vessels are remodeled. This leads to a change in arterial stiffness [18, 19] as well as to an increase in vessel wall thickness – that is initially confined to the intimal layer. This early vascular remodeling can be reliably measured by high resolution ultrasound (carotid intima-media-thickness, cIMT) as well as aortic (carotid-femoral) pulse wave velocity (PWV) [20, 21]. Both parameters are independent predictors for future cardiovascular events [22–25].

In order to shed light on the pathophysiologic link between MA and the increased risk for CVD, we strived to explore, if early vascular ageing (EVA) measured by high resolution ultrasound of the carotid arteries and carotid-femoral PWV differs between participants with MA, with other headaches and without headaches in a large cross-sectional cohort of healthy adolescents.

## Methods

### Participants

The EVA-Tyrol study is a community-based non-randomized controlled trial conducted in Tyrol (Austria) and in Bruneck (South-Tyrol, Italy) between May 2015 and September 2018 aiming to increase cardiovascular health in adolescents aged 14 years and above. The study protocol has been previously published elsewhere [26]. In brief, adolescents were recruited in schools and companies in North, East (Austria) and South Tyrol (Italy). The study population consisted of an intervention group and a control group. The 1573 participants of the intervention group were aged 14 to 17 years (mean age 15.99 years (standard deviation (SD) ± 0.94)) at the baseline examination and 1000 of those were re-examined after receiving health promotion program in a 2 year follow-up at a mean age of 17.71 years (± 0.91)). Further 529 participants aged 16 to 19 years (mean age 17.83 years (± 0.89)) from different schools and companies served as a control group.

**Fig. 1:**
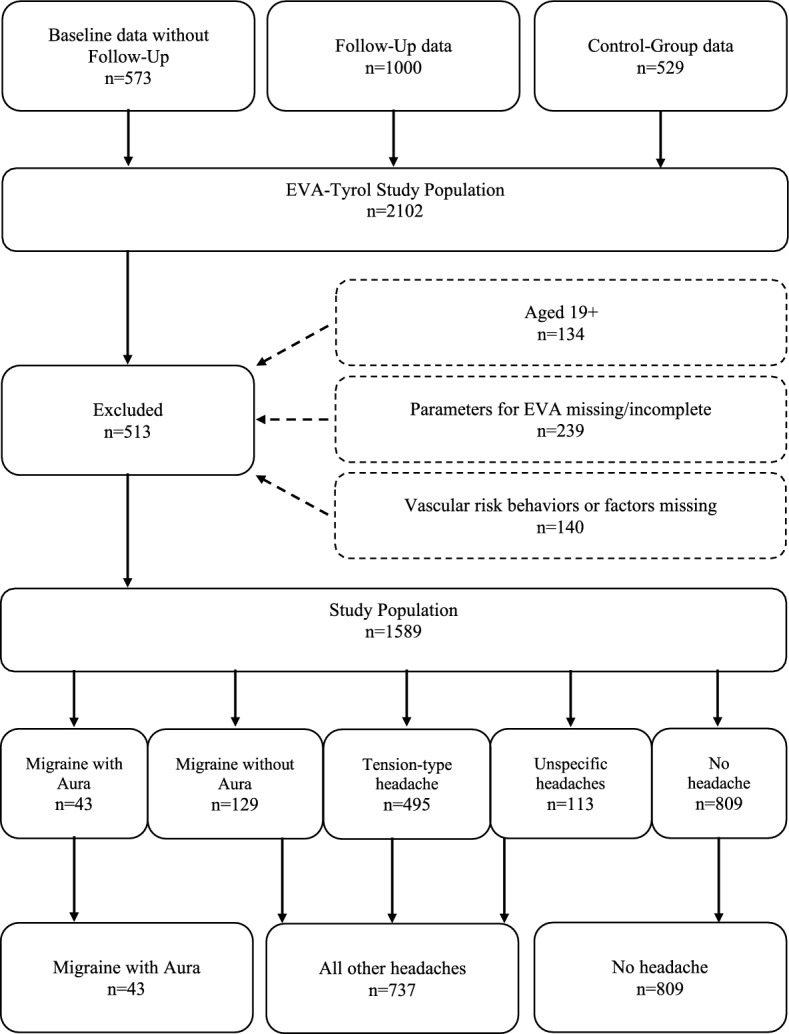
Flow-chart of study population, prevalence of headaches and group allocation

We included all adolescents aged 14 to 19 years with complete information on headache classification, sex, age, systolic blood pressure, body length, weight, physical activity, smoking habits, alcohol consumption, aspartate-aminotransferase (ASAT), blood-glucose level, high-density lipoprotein (HDL)- and low-density lipoprotein (LDL)-cholesterol. Data was used from the follow-up examination of the intervention group and from the control group. In case participants of the intervention group did not participate in the follow-up examination baseline data was used (overview in Fig. 1).

An informed consent was signed by all participants and if legal age (18 years) was not reached, additionally by a legal representative. EVA-Tyrol is registered on ClinicalTrials.gov 29/04/2019 (NCT03929692).

### Headache-survey

Primary headaches were classified in accordance with the International Classification of Headache Disorders 3^rd^ edition (ICHD-3) [27] in a structured face-to-face physician interview as MA, migraine without aura and tension-type headaches. If headaches did not fulfill the criteria of any primary headache, they were classified as unspecific headaches. If participants had MA combined with other headaches or fulfilled the criteria for probable MA, they were analyzed in the MA group only. As MA shows the highest absolute and relative risk for CVD, we compared MA against all other headaches (including migraine without aura) and against the no headache group. Headache characteristics and frequency as well as a detailed description of medication use in the EVA-Tyrol study population were priorly published elsewhere [28].

### Physical examination

Body length was measured using a portable stadiometer, body weight with a calibrated, metric balance. Systolic and diastolic blood pressure were calculated as the mean of 3 measurements on both arms in a sitting position, recorded after a 5‐minute seated rest (automated oscillometric device OMRON M4‐I, Omron Healthcare, Lake Forest, Illinois, United States of America).

### Blood sample collection and analysis

Plasma samples were drawn in the morning after an overnight fast and were immediately stored in cooling boxes at approximately 4°C before direct transfer to the Central Institute for Medical and Chemical Laboratory Diagnostics of Innsbruck University Hospital. Analyses were performed in a Cobas8000® (Roche). HDL- and LDL-cholesterol were measured using an enzymatic color essay, ASAT was analyzed using an enzyme spectrophotometry method and blood-glucose level was determined with a hexokinase method. Further parameters and measurement methods were published elsewhere [14, 26].

### Assessment of lifestyle and vascular risk factors

Lifestyle risk factors were assessed in a structured face-to-face interview by a specially trained medical doctor with a standardized questionnaire adapted from the Health Behavior in School-aged Children Survey Study [29], Bruneck Study and the Atherosclerotic Risk Factors in Male and Female Youngsters (ARMY-, ARFY-) Study [12, 13, 30]. Participants were classified as smokers if they regularly smoked at least one cigarette per week. Weekly consumption of the most common alcoholic beverages was inquired during the interview and was computed as average alcohol consumption in grams per week. Physical activity was assessed as moderate- or vigorous-intensity sports (i.e., leading to an increase in heart rate and/or sweating) in minutes per day. Family history of CVD was defined as the occurrence of an heart attack or stroke in family members aged less then 65 years (female) or 55 years (male).

### Pulse-wave-velocity (PWV)

PWV is a marker for aortic stiffness, with higher values indicating increased aortic stiffness as a parameter for accelerated EVA. PWV was measured twice in a supine position by a Complior-Analyze® (ALAMmedical, Paris, France) according to the manufacturer’s instructions and in accordance with previously validated protocols [31, 32]. In brief, ten consecutive pulse waves of artefact-free cardiac cycles were simultaneously recorded on the ipsilateral carotid and femoral arteries by applanation tonometry. Carotid-femoral PWV was calculated by measuring the surface travel distance and the mean wave transit time. The mean of two measurements was used.

### Carotid intima-media-thickness (cIMT)

We used high-resolution ultrasound to visualize the intima-media complex of the distal common carotid arteries on the far wall of both sides. The ultrasound protocol has been adapted from the ARMY and ARFY studies [12, 13], has been published previously [14, 26] and is concordant to the updated Mannheim cIMT and Plaque Consensus [33]. Measurements were conducted by experienced sonographers using a 6.0–13.0 MHz linear probe (GE 12L-RS) on a GE healthcare, Vivid q (both General Electric Healthcare, Chicago, Illinois, United States of America). cIMT measurement was done on stored images on three sites of the distal 4 cm segment of the left and right common carotid artery. Mean cIMT (cIMTmean) was calculated from all six measurements. The rater was blinded to the clinical characteristics of the participants.

### Statistics

The current analysis is a post-hoc secondary objective of the EVA Tyrol study. All statistical analyses were done with SPSS Version 27 (IBM, International Business Machines Corporation, Armonk, New York, United States of America). We focused on differences between MA and no headaches as well as MA and all other headaches. Univariate inter-group comparisons were done by the Mann-Whitney U test. We explored differences of PWV or cIMT between headache types with multivariable linear regression analysis. Linearity of all variables was investigated by scatterplots of residuals against fitted values, normality of residuals by Normal Q-Q-plots, and homoskedasticity by Spread-Location plots. The model was adjusted for variables with known effect on IMT or PWV from previous analyses [14] including sex, age, weight, body length, systolic blood pressure, physical activity, smoking habits, alcohol consumption, HDL- and LDL-cholesterol, ASAT and fasting blood-glucose-level [14, 26]. P-values of less than 0.05 were considered statistically significant. Further details on model building can be found in the supplemental material.

## Results

Of the 2102 participants of the EVA Tyrol study, 1968 (93.6%) were aged 14 to 19 years. Details on the participants aged 19 years and above as well as an analysis including participants of all ages can be found in the online supplement. After exlusion of those with missing information on IMT or PWV (*n* = 239) or vascular risk behaviours and headache characteristics (*n* = 140) 1589 (75.6%) served as study population for the current analysis (Fig. 1). 57.8% of the 1589 participants were female and 49.1% (*n* = 780) reported regular headaches. Females reported more often headaches than males (55.9 vs. 39.7%; *p* < 0.01). Frequency of headache classifications are shown in Fig. 1. Mean (SD) age was 16.8 (± 0.9) years. 5.5% (*n* = 43) of all reported headaches were MA, thereof 67.4% were female. There was a significant difference in sex and ASAT (*p* < 0.05) between participants with MA and no headaches. Systolic blood pressure, alcohol-consumption and on demand medication use differed significantly (*p* < 0.05) between MA and all other headaches. None of the participants did use prophylactic medication and 80.6% used on-demand medication (77.4% NSARs and 3.2% Tiptanes) for their MA. Population characteristics are given in Table 1.

**Table 1:** Characteristics of the study population according to the type of headaches (MA, all other headaches and no headaches)

	Migraine with aura	Other headaches	No headache	Total
	*N* = 43	*N* = 737	*N* = 809	*N* = 1589
Sex (female)	67.4%	65.7%	50.1%	57.8%
Age as completed years (SD)	16.9 (± 0.8)	16.9 (± 0.9)	16.8 (± 1.0)	16.8 (± 0.9)
Body length in cm (SD)	171.87 (± 7.9)	170.95 (± 8.9)	172.97 (± 9.1)	172.0 (± 9.0)
Weight in kg (SD)	65.1 (± 10.5)	65.4 (± 12.3)	66.3 (± 12.4)	65.8 (± 12.3)
Systolic blood pressure in mmHg (SD)	124.3 (± 11.2)	120.7 (± 12.0)	122.9 (± 12.6)	121.9 (± 12.3)
Diastolic blood pressure in mmHg (SD)	73.2 (± 8.5)	71.1 (± 7.8)	70.5 (± 7.9)	70.9 (± 7.9)
Family history of CVD	14.6%	13.3%	14.3%	13.8%
Physical activity: minutes/day (SD)	47.6 (± 33.4)	42.4 (± 33.5)	54.0 (± 42.4)	48.4 (± 38.7)
Smoking habits (yes)	23.3%	30.9%	25.3%	27.9%
Alcohol in gram/week (SD)	77.4 (± 76.6)	59.4 (± 74.0)	62.6 (± 79.4)	61.5 (± 76.9)
ASAT in U/l (SD)	20.5 (4.9)	22.7 (± 15.0)	23.8 (± 9.1))	23.2 (± 12.2)
Blood-glucose in mg/dl (SD)	77.4 (± 8.5)	76.7 (± 10.0)	77.7 (± 9.4)	77.2 (± 9.6)
HDL-cholesterol in mg/dl (SD)	59.3 (± 13.8)	57.7 (± 13.0)	57.9 (± 13.3)	57.8 (± 13.2)
LDL-cholesterol in mg/dl (SD)	95.8 (± 27.7)	96.5 (± 25.5)	93.9 (± 28.0)	95.2 (± 26.9)
**Headache characteristics**				
Frequency in days per month (SD)	6.6 (± 7.1)	5.8 (± 7.0)	n.a	5.7 (± 6.9)
On demand medication	80.6%	47.9%	n.a. %	49.8%
**Early vascular Ageing (EVA)**				
Pulse-Wave-Velocity in m/s (SD)	6.17 (± 0.85)	6.06 (± 0.82)	6.15 (± 0.95)	6.11 (± 0.89)
Intima-Media-Thickness in µm (SD)	411.3 (± 43.5)	410.9 (± 46.0)	421.6 (± 48.4)	416.4 (± 47.4)

Mean PWV (SD) ranged between 6.06 m/s (± 0.82) (all other headaches) and 6.17 m/s (± 0.85) (MA). None of MA, 0.1% of all other headaches and 0.5% of no headache had a relevantly increased PWV over 10 m/s.

Mean cIMT (SD) variated between 410.9 µm (± 46.0) (all other headaches) and 421.6 µm (± 48.4) (no headache). None of our participants showed an atherosclerotic plaque formation (maximal cIMT was 640 µm).

No differences in PWV or cIMT could be found between participants with MA and without headache or with other headache or when compared to both groups in univariable as well as multivariable linear regression analysis adjusted for age, sex, weight, blood pressure (supplemental material) or sex, age, systolic blood pressure, body length, weight, physical activity, smoking habits, alcohol consumption, ASAT, blood-glucose, HDL- and LDL-cholesterol (Table 2). The resultes remained essentially unaltered when diastolic instead of systolic blood pressure was entered to the model or if the model was supplemented by CVD-family history, the use of on-demand medication and headache frequency in days per month (results not shown).

**Table 2:** Comparisons of migraine with aura against no headache, all other headaches or both for PWV and cIMT (*n* = 1589)

	**Univariat Mann-Whitney U**		**Multivariable**^**a**^** Linear Regression**			
	z	*p*-value	Beta	*p*-value	95% Confidence Intervall	
					Lower	Upper
**PWV**						
Migraine with aura against no headache	-0.192	0.848	0.005	0.889	-0.298	0.259
Migraine with aura against all other headaches	-0.562	0.574	-0.012	0.734	-0.285	0.201
Migraine with aura against all other (other headaches and no headache)	-0.373	0.709	-0.005	0.842	-0.283	0.231
**cIMT**						
Migraine with aura against no headache	-1.396	0.163	-0.034	0.300	-6.700	21.742
Migraine with aura against all other headaches	-0.109	0.913	0.012	0.726	-11.301	16.207
Migraine with aura against all other (other headaches and no headache)	-0.687	0.492	0.019	0.439	-8.308	19.158

Two-sided *p*-values for Univariat Mann-Whitney U

^a^Linear regression, covariates in the model: sex, age, systolic blood pressure, body length, weight, physical activity, smoking habits, alcohol consumption, ASAT, blood-glucose, HDL- and LDL-cholesterol. These variables were entered as constant predictors

## Discussion

We present a large study comparing early signs of vascular ageing in an unselected large cohort of adolescents with MA, with other headaches and without headache. In summary, our data indicate that there are no relevant differences in carotid-femoral PWV or cIMT in young patients with MA, other headaches or no headaches, respectively.

Almost all previous research on this topic was conducted in adults. Even though smaller case-control studies yielded conflicting results with an increased PWV [34–36] and decreased [37] or increased [38, 39] markers of subclinical atherosclerosis in patients with MA, larger case-control [40] or cohort studies [41–43] could not demonstrate differences in PWV or subclinical atherosclerosis.

So far, only two case-control studies have addressed the present research question in children and adolescents. In the one study, cIMT was increased in 57 migraine patients (aged 5–17 years) when compared to 47 healthy controls, even though no differences in vascular risk profile were seen [44]. In the other study on the contrary, IMT was lower and flow-mediated dilatation of the brachial artery – another marker of vascular dysfunction – was higher (i.e. better) in 21 patients (aged 5–20 years) with migraine and 20 patients with migraine and history of syncope compared to 30 healthy controls [45]. No study has previously evaluated PWV in children with headaches.

However, the pediatric population is especially suited to address our research question. Even though cIMT was in the normal range in every single study participant and PVW was in the normal range in 99.5% of study participants, we – as well as others – have shown that vascular risk behaviors and factors are already associated with both parameters of vascular health [12–14, 46–48]. The association between MA and ischemic stroke is strongest in adults even without a cardiovascular risk profile [49, 50]. A recent publication with 5691 participants confirmed a higher CVD-risk (OR 2.77) for patients with migraine or severe headaches [51]. Furthermore a Danish population based study with 51,032 migraine patients and 510,320 peope from the general population showed an higher hazard ration for migraine and myocardial infarction (Hazard ratio 1.49), ischaemic stroke (HR 2.26) or haemorrhagic stroke (HR 1.94) [52]. Migraine with aura had an higher incidence rate of major CVD in a study with 27,858 health professionals (3.36 vs 2.11 per 1000 person years) [53]. Possible associations between EVA and MA might be confounded in adults on the background of a longer duration and higher prevalence of traditional cardiovascular risk factors. Therefore, a cohort of adolescents with a relatively short exposure to classical cardiovascular risk factors would be very sensitive to detect a possible link between MA and EVA.

When interpreting our results, one must keep in mind, that we only evaluated early structural vessel wall changes in the common carotid artery and that carotid-femoral PWV primarily measures aortic stiffness [25, 47]. Information on other conditions that have been proposed as possible link between MA and CVD like the presence of a patent foramen ovale (PFO), small vessel disease or autonomic dysfunction is not available in our cohort. The prevalence of PFO is higher in both – patients with MA and cryptogenic stroke [54, 55], yet the association between MA and stroke is independent of the presence of PFO [56]. Furthermore, we cannot rule out that MA is associated with smaller vessel dysfunction or even more specific alterations in small brain vessel function, as we focused on large and proximal vessels in our study. In adults small vessel disease are often characterized by early stroke and migraine [57]. In addition, the cumulative number of MA attacks is generally low in adolescents because of the much shorter disease duration when compared to adults and due to the fact that MA is not yet a common disorder in our young population (2.7% in our cohort) and therefore EVA as a possible consequence of cumulative repeated MA attacks might yet be below the level of detection in adolescents. Also, our study is not powered to explore effects of different types of aura, aura duration and family history for MA on EVA. Furthermore, our analysis might – in part – be subject to a misclassification of headache bias. Even though we performed a classification of headache syndromes based on the ICHD-3 classification in a structured face-to-face physician interview, adolescents with a relatively short headache history might have troubles to adequately describe their headache characteristics. Yet, we would consider the diagnosis of MA can reliably be established in this age group. Also, participants that do have an endothelial dysfunction predisposing for MA might not have developed migraine so far, which could further attenuate the statistical power of its correlation at this young age. Finally, we cannot completely rule out a selection bias. Adolescents with known unfavorable risk profile or primary headache disorder might not have been more or less likely to participate in our study, yet, we would not expect that this alters the (non)association observed.

The special strengths of our study are the community-based cohort setting, the large sample size, the high quality of the headache classification by a structured physician guided interview and the exact evaluation of EVA as well as of cardiovascular risk factors and behaviors.

## Conclusion

In line with previous large-scale studies in adults, we could not demonstrate relevant associations of MA with markers of arterial stiffness or subclinical atherosclerosis in adolescents making EVA an unlikely pathophysiological link between MA and CVD.

## Supplementary Information

**Additional file 1: Table S1.** Comparative characteristics migraine with aura. **Table S2.** Comparative characteristics no headache. **Table S3.** Comparative characteristics all other headaches. **Table S4.** Comparative characteristics total. **Table S5.** Comparisons of migraine with aura against no headache, all other headaches or both for PWV and cIMT with a reduced number of co-variates for our peer group aged 14 to 19 years. **Table S6.** Comparisons of migraine with aura against no headache, all other headaches or both for PWV and cIMT for all available participants (aged 14 to 23 years). **Table S7.** Comparisons of migraine with aura against no headache, all other headaches or both for PWV and cIMT with a reduced number of co-variates for all available participants (aged 14 to 23 years).

## Data Availability

Request for data shall be addressed to the corresponding authors.

## Abbreviations

EVA: Early vascular ageing
WHO: World Health Organization
MA: Migraine with aura
CVD: Cardiovascular diseases
TIA: Transient ischemic attack
cIMT: Carotid intima-media-thickness
PWV: Pulse-wave velocity
SD: Standard deviation
ASAT: Aspartate-aminotransferase
HDL: High-density lipoprotein
LDL: Low-density lipoprotein
ICHD-3: International classification of headache D’disorders 3^rd^ edition
ARMY: Atherosclerotic risk factors in male youngsters
ARFY: Atherosclerotic risk factors in female youngsters
PFO: Patent foramen ovale
