# Supplementary material for: Early Vascular Ageing in adolescents with migraine with aura: a community-based study
Source: BMC Cardiovasc Disord. 2023 Aug 1;23:384. doi: 10.1186/s12872-023-03409-2 (PMC10394858; doi:10.1186/s12872-023-03409-2)
Supplement: Supplementary file 1 — Additional file 1: Table S1. Comparative characteristics migraine with aura. Table S2. Comparative characteristics no headache. Table S3. Comparative characteristics all other headaches. Table S4. Comparative characteristics total. Table S5. Comparisons of migraine with aura against no headache, all other headaches or both for PWV and cIMT with a reduced number of co-variates for our peer group aged 14 to 19 years. Table S6. Comparisons of migraine with aura against no headache, all other headaches or both for PWV and cIMT for all available participants (aged 14 to 23 years). Table S7. Comparisons of migraine with aura against no headache, all other headaches or both for PWV and cIMT with a reduced number of co-variates for all available participants (aged 14 to 23 years). [file 12872_2023_3409_MOESM1_ESM.docx]

**Early Vascular Ageing in adolescents with migraine with aura: a community-based study**

B Bernar (^1;2^), N Gande (^3^), K Stock (^3;4^), A Staudt (^3^), R Pechlaner (^2^), C Hochmayr (^3^), K Kaltseis (^2^), B Winder (^3;5;6^), SJ Kiechl (^2;6;7^), G Broessner (^2^), R Geiger (^4^), S Kiechl (^2;6^), Early Vascular Ageing (EVA) Tyrol Study Group ^Ω^, U Kiechl-Kohlendorfer*(^3^), M Knoflach*(^2;6^)

1. Medical University of Innsbruck, Dept. of Pediatrics, Pediatrics I, Innsbruck, Austria
2. Medical University of Innsbruck, Dept. of Neurology, Innsbruck, Austria
3. Medical University of Innsbruck, Dept. of Pediatrics, Pediatrics II, Innsbruck, Austria
4. Medical University of Innsbruck, Dept. of Pediatrics, Pediatrics III, Innsbruck, Austria
5. Academic Teaching Hospital, Landeskrankenhaus Feldkirch, Feldkirch, Austria
6. VASCage, Research Centre on Vascular Ageing and Stroke, Innsbruck, Austria.
7. Department of Neurology, Hochzirl-Natters Hospital, Zirl, Austria

*Contributed equally

Ω The full list of consortium members can be found at the end of the manuscript

**Supplemental Material**

**Statistics:**

Models were fitted using data from 1589 (Migraine with aura against all other) adolescents and included 12 predictors, resulting on average in more than 130 subjects per parameter. To estimate the effect of migraine with aura against those without headache (n = 780), one parameter was fitted using 43 data points. Although there is no definitive consensus on the necessary number of subjects per parameter (compare eg. Vittinghoff. Relaxing the rule of ten events per variable in logistic and Cox regression; or Harrell. Regression Modeling Strategies: With Applications to Linear Models, Logistic and Ordinal Regression, and Survival Analysis. Second ed. Cham, Switzerland: Springer International Publishing; 2015.), the traditional rule of thumb is that 10 subjects per parameter are required, and the amount of data available in our study would be considered sufficient to reliably estimate effects according to most standards.

With regard to linear model assumptions, linearity was investigated by scatterplots of residuals against fitted values, normality of residuals by Normal Q-Q-plots, and homoskedasticity by Spread-Location plots. Model assumptions were met in all cases.

**Excluded data:**

We have 2102 participants aged 14 to 23 years, we excluded 134 participants due to their age (19+), thereof in 124 headache classification was available. In 120 of 134 all data was complete. 8 (6.5%) had migraine with aura, 56 (54%) had no headache and 60 (48%) had other headaches.

After excluding 402/2102 due to missing data, 1589 were aged 14 to 19 years and 111 were aged 19 to 23 years. Including all participants with complete data: 863 had no headaches, 49 had migraine with aura and 788 had other headaches. Comparing both groups (14 to 19 and 14 to 23 years): 50.9% vs 50.8% had no headache, 2.7% vs 2.9% had migraine with aura and 46.4% vs 46.4 % had other headaches, there were no significant differences between both groups. Headache characteristics did not differ between included and excluded participants, comparative characteristics of our studied group (14 to 19 years) and all available participants (14 to 23 years) can be found in table S1-4.

Comparing participants with complete data:

Mean PWV was, as expected significantly higher in the 19+ group (6.11 ±0.89 vs 6.42 ±0.76, p<0.001).PWV increased from 5.85 ±1.55 (14years) to 6.17 ±0.86 (18 years) to 6.42 ±0.76 (19+).

Mean cIMT was (non-significantly) higher in the 19+ group (416µm ±47 vs 417µm ±47, p=0.539).

**Different multivariable models:**

In order to avoid a bias by over-fitting of our model, we evaluated different multivariable models. Reducing the number of covariates to those with significant impact on IMT and/or PWV (age, sex, weight, blood pressure) did not change the results (results are shown in table S5-7).

|  | Migraine with aura  Aged 14 to 19 years | Migraine with aura  Aged 14 to 23 years  (all available participants) |
| --- | --- | --- |
| Sex (female) | 67.4 % | 65.3 % |
| Age as completed years (SD) | 16.9 (±0.8) | 17.1 (±1.1) |
| Body length in cm (SD) | 171.87 (±7.9) | 172.22 (±8.1) |
| Weight in kg (SD) | 65.1 (±10.5) | 65.1 (±10.1) |
| Systolic blood pressure in mmHg (SD) | 124.3 (±11.2) | 123.6 (±10.8) |
| Diastolic blood pressure in mmHg (SD) | 73.2 (±8.5) | 72.7 (±8.7) |
| Family history of CVD | 14.6 % | 14.9 % |
| Physical activity: minutes/day (SD) | 47.6 (±33.4) | 46.1 (±32.0) |
| Smoking habits (yes) | 23.3 % | 24.5 % |
| Alcohol in gram/week (SD) | 77.4 (±76.6) | 73.6 (±74.0) |
| ASAT in U/l (SD) | 20.5 (4.9) | 20.5 (±4.7) |
| Blood-glucose in mg/dl (SD) | 77.4 (±8.5) | 77.4 (±8.4) |
| HDL-cholesterol in mg/dl (SD) | 59.3 (±13.8) | 58.6 (±13.2) |
| LDL-cholesterol in mg/dl (SD) | 95.8 (±27.7) | 95.2 (±26.7) |
|  |  |  |
| **Headache characteristics** |  |  |
| Frequency in days per month (SD) | 6.6 (±7.1) | 6.5 (±6.7) |
| On demand medication | 80.6 % | 78.4 % |
|  |  |  |
| **Early vascular Ageing (EVA)** |  |  |
| Pulse-Wave-Velocity in m/s (SD) | 6.17 (±0.85) | 6.28 (±0.90) |
| Intima-Media-Thickness in µm (SD) | 411.3 (±43.5) | 412.5 (±42.5) |

*Table S1: Comparative characteristics* ***migraine with aura***

|  | No headache  Aged 14 to 19 years | No headache  Aged 14 to 23 years  (all available participants) |
| --- | --- | --- |
| Sex (female) | 50.1 % | 48.8 % |
| Age as completed years (SD) | 16.8 (±1.0) | 16.9 (±1.1) |
| Body length in cm (SD) | 172.97 (±9.1) | 173.17 (±9.1) |
| Weight in kg (SD) | 66.3 (±12.4) | 66.8 (±12.7) |
| Systolic blood pressure in mmHg (SD) | 122.9 (±12.6) | 123.1 (±12.6) |
| Diastolic blood pressure in mmHg (SD) | 70.5 (±7.9) | 70.5 (±7.9) |
| Family history of CVD | 14.3 % | 14.6 % |
| Physical activity: minutes/day (SD) | 54.0 (±42.4) | 53.9 (±41.7) |
| Smoking habits (yes) | 25.3 % | 25.8 % |
| Alcohol in gram/week (SD) | 62.6 (±79.4) | 63.3 (±79.7) |
| ASAT in U/l (SD) | 23.8 (±9.1)) | 23.7 (±9.0) |
| Blood-glucose in mg/dl (SD) | 77.7 (±9.4) | 77.8 (±9.4) |
| HDL-cholesterol in mg/dl (SD) | 57.9 (±13.3) | 57.6 (±13.2) |
| LDL-cholesterol in mg/dl (SD) | 93.9 (±28.0) | 93.9 (±27.9) |
|  |  |  |
| **Headache characteristics** |  |  |
| Frequency in days per month (SD) | n.a. | n.a. |
| On demand medication | n.a. % | n.a. % |
|  |  |  |
| **Early vascular Ageing (EVA)** |  |  |
| Pulse-Wave-Velocity in m/s (SD) | 6.15 (±0.95) | 6.16 (±0.95) |
| Intima-Media-Thickness in µm (SD) | 421.6 (±48.4) | 421.7 (±48.1) |

*Table S2: Comparative characteristics* ***no headache***

|  | All other headaches  Aged 14 to 19 years | All other headaches  Aged 14 to 23 years  (all available participants) |
| --- | --- | --- |
| Sex (female) | 65.7 % | 64.2 % |
| Age as completed years (SD) | 16.9 (±0.9) | 17.1 (±1.1) |
| Body length in cm (SD) | 170.95 (±8.9) | 171.18 (±9.0) |
| Weight in kg (SD) | 65.4 (±12.3) | 65.9 (±12.6) |
| Systolic blood pressure in mmHg (SD) | 120.7 (±12.0) | 120.9 (±12.1) |
| Diastolic blood pressure in mmHg (SD) | 71.1 (±7.8) | 71.2 (±7.8) |
| Family history of CVD | 13.3 % | 13.5 % |
| Physical activity: minutes/day (SD) | 42.4 (±33.5) | 43.3 (±36.6) |
| Smoking habits (yes) | 30.9 % | 31.1 % |
| Alcohol in gram/week (SD) | 59.4 (±74.0) | 59.1 (±73.7) |
| ASAT in U/l (SD) | 22.7 (±15.0) | 22.8 (±14.7) |
| Blood-glucose in mg/dl (SD) | 76.7 (±10.0) | 77.0 (±10.2) |
| HDL-cholesterol in mg/dl (SD) | 57.7 (±13.0) | 57.4 (±13.0) |
| LDL-cholesterol in mg/dl (SD) | 96.5 (±25.5) | 96.5 (±25.5) |
|  |  |  |
| **Headache characteristics** |  |  |
| Frequency in days per month (SD) | 5.8 (±7.0) | 5.7 (±6.9) |
| On demand medication | 47.9 % | 48.5 % |
|  |  |  |
| **Early vascular Ageing (EVA)** |  |  |
| Pulse-Wave-Velocity in m/s (SD) | 6.06 (±0.82) | 6.08 (±0.81) |
| Intima-Media-Thickness in µm (SD) | 410.9 (±46.0) | 410.9 (±46.3) |

*Table S3: Comparative characteristics* ***all other headaches***

|  | Total  Aged 14 to 19 years | Total  Aged 14 to 23 years  (all available participants) |
| --- | --- | --- |
| Sex (female) | 57.8 % | 56.4 % |
| Age as completed years (SD) | 16.8 (±0.9) | 17.0 (±1.1) |
| Body length in cm (SD) | 172.0 (±9.0) | 172.2 (±9.1) |
| Weight in kg (SD) | 65.8 (±12.3) | 66.3 (±12.6) |
| Systolic blood pressure in mmHg (SD) | 121.9 (±12.3) | 122.1 (±12.3) |
| Diastolic blood pressure in mmHg (SD) | 70.9 (±7.9) | 70.9 (±7.9) |
| Family history of CVD | 13.8 % | 14.1 % |
| Physical activity: minutes/day (SD) | 48.4 (±38.7) | 48.8 (±39.5) |
| Smoking habits (yes) | 27.9 % | 28.2 % |
| Alcohol in gram/week (SD) | 61.5 (±76.9) | 61.7 (±76.8) |
| ASAT in U/l (SD) | 23.2 (±12.2) | 23.2 (±11.9) |
| Blood-glucose in mg/dl (SD) | 77.2 (±9.6) | 77.4 (±9.8) |
| HDL-cholesterol in mg/dl (SD) | 57.8 (±13.2) | 57.5 (±13.1) |
| LDL-cholesterol in mg/dl (SD) | 95.2 (±26.9) | 95.1 (±26.8) |
|  |  |  |
| **Headache characteristics** |  |  |
| Frequency in days per month (SD) | 5.7 (±6.9) | 5.6 (±6.9) |
| On demand medication | 49.8 % | 50.4 % |
|  |  |  |
| **Early vascular Ageing (EVA)** |  |  |
| Pulse-Wave-Velocity in m/s (SD) | 6.11 (±0.89) | 6.13 (±0.89) |
| Intima-Media-Thickness in µm (SD) | 416.4 (±47.4) | 416.4 (±47.4) |

*Table S4: Comparative characteristics* ***total***

|  | **Multivariable* Linear Regression** | | | | | | | | | |
| --- | --- | --- | --- | --- | --- | --- | --- | --- | --- | --- |
|  | | | Beta | | p-value | | | 95% Confidence Intervall | | |
|  | | |  | | | | Lower | | | Upper |
| **PWV**  Migraine with aura against no headache | | 0.008 | | 0.816 | | -0.244 | | | 0.309 | |
| Migraine with aura against all other headaches | | -0.012 | | 0.719 | | -0.285 | | | 0.197 | |
| Migraine with aura against all other (other headaches and no headache) | | -0.006 | | 0.803 | | -0.289 | | | 0.224 | |
| **cIMT**  Migraine with aura against no headache | | -0.034 | | 0.307 | | -21.579 | | | 6.800 | |
| Migraine with aura against all other headaches | | 0.003 | | 0.932 | | -13.148 | | | 14.337 | |
| Migraine with aura against all other (other headaches and no headache) | | 0.015 | | 0.541 | | -9.484 | | | 18.064 | |

** linear regression, covariates in the model:* ***sex, age, systolic blood pressure, weight***

*Table S5: Comparisons of migraine with aura against no headache, all other headaches or both for PWV and cIMT* ***with a reduced number of co-variates for our peer group aged 14 to 19 years.***

|  | **Univariat Mann-Whitney U** | | | | **Multivariable* Linear Regression** | | | | |
| --- | --- | --- | --- | --- | --- | --- | --- | --- | --- |
|  | | | z | p-value | Beta | p-value | | 95% Confidence Intervall | |
|  | | |  | |  | | Lower | | Upper |
| **PWV** | | | | | | | | | |
| Migraine with aura against no headache | | -0.825 | | 0.409 | 0.026 | 0.413 | -0.152 | | 0.369 |
| Migraine with aura against all other headaches | | -1.214 | | 0.225 | -0.039 | 0.239 | -0.360 | | 0.090 |
| Migraine with aura against all other (other headaches and no headache) | | -1.026 | | 0.305 | -0.022 | 0.335 | -0.357 | | 0.122 |
| **cIMT** | | | | | | | | | |
| Migraine with aura against no headache | | -1.276 | | 0.202 | -0.026 | 0.414 | -18.758 | | 7.725 |
| Migraine with aura against all other headaches | | -0.335 | | 0.738 | 0.002 | 0.952 | -12.566 | | 13.364 |
| Migraine with aura against all other (other headaches and no headache) | | -0.514 | | 0.607 | 0.012 | 0.599 | -9.418 | | 16.332 |

** linear regression, covariates in the model: sex, age, systolic blood pressure, body length, weight, physical activity, smoking habits, alcohol consumption, ASAT, blood-glucose, HDL- and LDL-cholesterol. These variables were entered as constant predictors.*

*Two-sided p-values for Univariat Mann-Whitney U*

*Table S6: Comparisons of migraine with aura against no headache, all other headaches or both for PWV and cIMT* ***for all available participants (aged 14 to 23 years)***

|  | **Multivariable* Linear Regression** | | | | | | | | | |
| --- | --- | --- | --- | --- | --- | --- | --- | --- | --- | --- |
|  | | | Beta | | p-value | | 95% Confidence Intervall | | | |
|  | | |  | | | Lower | | | | Upper |
| **PWV**  Migraine with aura against no headache | | 0.030 | | 0.342 | | | | -0.134 | 0.385 | |
| Migraine with aura against all other headaches | | -0.041 | | 0.218 | | | | -0.363 | 0.083 | |
| Migraine with aura against all other (other headaches and no headache)  **cIMT** | | -0.024 | | 0.299 | | | | -0.365 | 0.112 | |
| Migraine with aura against no headache | | -0.026 | | 0.406 | | | | -18.816 | 7.624 | |
| Migraine with aura against all other headaches | | -0.006 | | 0.851 | | | | -14.199 | 11.724 | |
| Migraine with aura against all other (other headaches and no headache) | | 0.009 | | 0.707 | | | | -10.438 | 15.390 | |

** linear regression, covariates in the model:* ***sex, age, systolic blood pressure, weight***

*Table S7: Comparisons of migraine with aura against no headache, all other headaches or both for PWV and cIMT* ***with a reduced number of co-variates for all available participants (aged 14 to 23 years)***
